# The Physiological Action and Therapeutic Value of Sclerotic Acid

**Published:** 1880-05

**Authors:** 


The Physiological Action and Therapeutic Value of Sclerotic Acid.—Sclerotic acid is probably the active principle of ergot of rye. It was discovered in 1877 by Dragendorff, of Dorpat, and is an amorphous, cinnamon-brown substance, of the empirical formula $C_{12}H_{19}NO_9$, hygroscopic, and readily soluble in water. Good ergot contains about four or five per cent. of it in combination with bases. It is a very feeble acid, and in the presence of calcic carbonate only causes an evolútion of carbonic acid when warmed. With sodium, however, it forms a stable sclerotinate. With phospho-molybdate of ammonia its aqueous solution gives a dark green, with tannin a reddish, percipitate. So much for its chemistry. Its physiological properties have lately undergone very careful investigation by Nikitin, in Professor Rossbach's laboratory at Wurburg (Wurzburger Phys. Med. Verhand., xiii; Centralblatt Med. Wiss., No. 42, 1879.) He finds that it is so rapidly excreted by the kidneys in warm-blooded animals that not a trace can be detected in from forty to forty-eight hours after its administration. Very large doses cause death by general paralysis; and in warm-blooded animals their immediate effect is a depression of temperature of from 1° to 3° Cent., which continues until death. Professor Binz (Grundzuge der Arnzneimittellehre-Sechste Auflage, S. 53), states that 0.01 gramme sclerotic acid gradually brought on general paralysis in a middle-sized specimen of Rana esculenta, but that the next day the creature was again well and lively. Nikitin sums up the action of sclerotic acid and its sodium salt as follows:—They both possess the physiological and therapeutic properties of ergot itself, but the sclerotinate is a weaker drug than the acid. Both chiefly act on the central nervous system. Frogs are very sensitive to the acid; among mammals, carnivora are more effected by than herbivora. In frogs the reflex irritability of the spinal cord is completely abolished; in warm-blooded animals it is never quite lost even just before death. The acid only paralyzes the sensory if brought into direct contact with their peripheral ends; the motor nerves as well as the striped muscles are unaffected by it. In mammals the heart is not influenced even by relatively large doses. The blood-pressure is only permanently reduced by large doses. At death the respiration ceases before the heart. In mammals the acid accelerates the intestinal peristalsis; and it excites contraction both of the pregnant and non-pregnant uterus, preexisting contractions being intensified so that the organ assumes a paler tint. Nikitin has not observed any poisonous effect on the fœ *in utero* either from the acid or the sodium sclerotinate. From his experiments on animals, he calculates that an adult man weighing fifty kilogrammes would be killed by about ten grammes sclerotic acid. In any case it is as much less powerful poison than most of our officinal alkaloids. The ordinary dose which has been hitherto given by subcutaneous injection is, however, only 0.02 to 0.03 gramme two or three times daily. Sclorotic acid seems likely before long to partially replace ergot as a drug. It has the advantage of remaining indefinitely without loss of strength, if only kept in a dry place and undissolved. Its sodium salt Nikitin considers the best preparation for internal use in the human subject. Subcutaneous injection of either drug causes a "sharp biting" pain, which passes off in a few minutes. Von Ziemssen claims for sclerotic acid over ergotin that the former causes no inflammation at the seat of puncture.—*London (Eng.) Med. Times and Gazette.*

